# The ADHD Concomitant Difficulties Scale (ADHD-CDS), a Brief Scale to Measure Comorbidity Associated to ADHD

**DOI:** 10.3389/fpsyg.2016.00871

**Published:** 2016-06-14

**Authors:** Javier Fenollar-Cortés, Luis J. Fuentes

**Affiliations:** ^1^Departamento de Psicología Evolutiva y de la Educación, Facultad de Psicología, Universidad de MurciaMurcia, Spain; ^2^Departamento de Psicología Básica y Metodología, Universidad de MurciaMurcia, Spain

**Keywords:** attention-deficit/hyperactivity disorder, concomitant difficulties, screening, ADHD functional difficulties, academic achievement, problem-solving/management of time, quality of life

## Abstract

**Introduction:** Although the critical feature of attention-deficit/hyperactivity disorder (ADHD) is a persistent pattern of inattention and/or hyperactivity/impulsivity behavior, the disorder is clinically heterogeneous, and concomitant difficulties are common. Children with ADHD are at increased risk for experiencing lifelong impairments in multiple domains of daily functioning. In the present study we aimed to build a brief ADHD impairment-related tool -ADHD concomitant difficulties scale (ADHD-CDS)- to assess the presence of some of the most important comorbidities that usually appear associated with ADHD such as emotional/motivational management, fine motor coordination, problem-solving/management of time, disruptive behavior, sleep habits, academic achievement and quality of life. The two main objectives of the study were (i) to discriminate those profiles with several and important ADHD functional difficulties and (ii) to create a brief clinical tool that fosters a comprehensive evaluation process and can be easily used by clinicians.

**Methods**: The total sample included 399 parents of children with ADHD aged 6–18 years (*M* = 11.65; *SD* = 3.1; 280 males) and 297 parents of children without a diagnosis of ADHD (*M* = 10.91; *SD* = 3.2; 149 male). The scale construction followed an item improved sequential process.

**Results**: Factor analysis showed a 13-item single factor model with good fit indices. Higher scores on inattention predicted higher scores on ADHD-CDS for both the clinical sample (β = 0.50; *p* < 0.001) and the whole sample (β = 0.85; *p* < 0.001). The ROC curve for the ADHD-CDS (against the ADHD diagnostic status) gave an area under the curve (AUC) of.979 (95%, CI = [0.969, 0.990]).

**Discussion**: The ADHD-CDS has shown preliminary adequate psychometric properties, with high convergent validity and good sensitivity for different ADHD profiles, which makes it a potentially appropriate and brief instrument that may be easily used by clinicians, researchers, and health professionals in dealing with ADHD.

## Introduction

It is amply accepted that Attention-Deficit/Hyperactivity Disorder (hereafter ADHD) impacts negatively on social, academic, and occupational functioning (American Psychiatric Association, 2013). Therefore, early detection and treatment of the disease is of crucial interest in the clinical and educational domains (Sonuga-Barke et al., 2011). Although the critical feature of ADHD is a persistent pattern of inattention and/or hyperactivity/impulsivity behavior, the disorder is rather heterogeneous at multiple levels (Wåhlstedt et al., 2009), and concomitant difficulties are the rule rather than the exception. The relationship between ADHD clinical profile and some functional impairment could be of especial interest not only to ADHD diagnosis/assessment but also to clinical intervention. Children with ADHD are at increased risk of experiencing serious, lifelong impairments in multiple domains of daily functioning. They include cognitive, language, adaptive functioning, motor development, emotion, school performance, task performance, and medical/health risks (Barkley, 2006). In the following sections we briefly comment on the main comorbidities exhibited by children with an ADHD diagnosis.

### Emotional/Motivational Management

Several studies have highlighted some alterations in emotional processing (Anastopoulos et al., 2011; Classi et al., 2012), as well as higher sensitivity to reward and motivation impairments as core neurocognitive deficits associated to ADHD (e.g., Fosco et al., 2015; van Hulst et al., 2015). Importantly, both emotional and motivational difficulties also persist in adulthood (Retz et al., 2012; Jarrett, 2016). Accordingly, ADHD has been associated with risks of undergoing depression, negative self-concept and low self-esteem, persisting until adulthood (Edbom et al., 2006).

### Fine Motor Coordination

Research has also suggested primary deficits in motor coordination associated to ADHD (Goulardins et al., 2013). It has mainly been observed in tasks that require rather complex motor skills (Scharoun et al., 2013), affecting up to 30–50% of ADHD cases (Fliers et al., 2008). Motor difficulties have also been related to poor quality and quantity of handwriting in ADHD (Rosenblum et al., 2008; Brossard-Racine et al., 2011; Shen et al., 2012). Although these impairments have a negative impact on academic achievement (Fliers et al., 2008), researchers have paid little attention to this comorbid problem, and consequently it has usually been excluded from the ADHD assessment (Fliers et al., 2010). In fact, motor difficulties have been thought of as an entity separated from the attention deficit (Pitcher et al., 2003; Miyahara et al., 2006), and pharmacological interventions do not seem to produce any remarkable improvement in motor coordination of ADHD individuals (e.g., Bart et al., 2010).

### Problem-Solving/Management of Time

Time processing is also affected in ADHD as it is evidenced in both behavioral (Hwang et al., 2010; Zelaznik et al., 2012), and neuroimaging (Hart et al., 2012) studies. The representation of time is crucial not only for everyday functioning but also to make long-term life plans (see Noreika et al., 2013 for review). Related to the time perception deficit is the difficulty that children with ADHD experience in tasks that require order or sequence (Barkley and Murphy, 2006). Poor skills in the management of time may be on the basis of the poor problem solving strategies frequently shown by these children. Accordingly, training metacognitive skills has shown good results in ADHD children (Tamm et al., 2014).

### Disruptive Behavior

The oppositional defiant disorder (ODD) is the most common comorbid condition associated to ADHD during childhood (Steinhausen et al., 2006). Children with ADHD have an increased risk to develop disruptive behavior (Bendiksen et al., 2014), which extends into the adulthood period (e.g., Reimherr et al., 2013). ADHD is also considered a predictor of risky sexual behavior (Flory et al., 2006), romantic partner problems (Wymbs et al., 2011), legal problems (Ginsberg et al., 2010), and unemployment in adulthood (Kessler et al., 2006).

### Sleep Habits

Children and adults with ADHD may present some symptoms related to sleep disorders such as daytime sleep, insomnia, fractured sleep, restless legs syndrome and sleep-disordered breathing (for a review, see Yoon et al., 2012). Despite a relationship between ADHD and sleep disorders has been documented, whether such relationship is direct or indirect is still unclear (e.g., Accardo et al., 2012). Consequently, some authors have suggested including the assessment of children’s sleep habits as part of the ADHD clinical diagnosis routine (e.g., Spruyt and Gozal, 2011).

### Academic Achievement

The relationship between ADHD and poor academic achievement is well established (Loe and Feldman, 2007; Langberg et al., 2011; for a review, see Arnold et al., 2015; for longitudinal studies, see Barbaresi et al., 2007; Sayal et al., 2015). In fact, learning disorders affecting language, reading, and math are common comorbidities of children diagnosed with ADHD (DuPaul et al., 2014; Taanila et al., 2014). Also, symptoms of inattention, hyperactivity, and impulsivity with or without formal diagnosis of ADHD are related with poor academic and educational outcomes (Birchwood and Daley, 2012), specially with inattentiveness (Wu and Gau, 2013). Similarly, poor productivity and low self-management of homework are frequent in children with ADHD (Power et al., 2006).

### Quality of Life

Attention-deficit/hyperactivity disorder affects children and adolescents’ quality of life involving psychosocial, achievement, and self-evaluation domains (for a review, see Wehmeier et al., 2010). Families with an ADHD child show some functioning deficits regarding both economy (Harpin, 2005) and marital relations (Escobar et al., 2005). The divorce rate of parents with an ADHD child is higher compared with parents of children without ADHD (Wymbs et al., 2008; Kvist et al., 2013). Thus, quality of life is emerging as an important aspect of the comprehensive diagnosis of ADHD (Coghill, 2010).

### The Current Study

Here we are concerned with developing a brief scale that provides both clinicians and researchers with an appropriate complementary diagnostic tool that takes into account the diversity of symptoms and the heterogeneity of the ADHD disease. The scale is also meant to providing clinicians with an instrument that can help design more comprehensive therapeutic targets that include not only the core ADHD symptoms but also some important difficulties associated with the disease. But, what would such a scale, which is intended to assess non-ADHD specific features that simply co-occur with the disease, add to already existing ADHD screening instruments?

First, most existing ADHD rating scales show certain constrains regarding the ADHD assessment process (e.g., Snyder et al., 2006), which may lead many clinicians to recruit additional clinical information (Pelham et al., 2005; Posserud et al., 2014; Shemmassian and Lee, 2014). Second, despite there are some scales that assess comorbid difficulties associated with an ADHD diagnosis, (e.g., Conners 3; Conners, 2008), the majority of them are rather long, expensive and are not intended to be used for rather wide screening processes (e.g., in schools). In addition, they hardly assess the presence of comorbidity beyond some behavioral disruptive symptoms. While their relevance is undeniable diagnostic-wise, it is more questionable treatment-wise. Third, although there are some brief screening scales (e.g., Impairment Rating Scale, Fabiano et al., 2006; Child Behavior Checklist, Achenbach and Rescorla, 2001; Child and Adolescent Functional Assessment Scale Hodges and Wong, 1996), they have not been specifically designed to assess comorbidity associated to ADHD. In addition, the majority of the screening scales for ADHD are primarily based on core symptoms of the disorder (i.e., inattention and hyperactivity/impulsivity symptoms), which generates a decrease in the sensibility of the scales due to subclinical heterogeneity (e.g., Ullebø et al., 2011).

In the present study we aimed to build a brief ADHD impairment-related tool, the ADHD Concomitant Difficulties Scale (hereafter ADHD-CDS), that serves two main functions: (i) to discriminate some profiles that present several and important ADHD functional difficulties; and (ii) to foster a comprehensive evaluation process that can be easily used by both clinicians and researchers. The aim of the ADHD-CDS was to assess the presence of some of the most important comorbidities, previously described, that usually appear associated to ADHD. It is important to highlight that the aforementioned deficits are not attributable directly to ADHD; they just co-occur frequently with the disease and therefore it cannot permit establishing any causal relation.

## Materials and Methods

### Participants

A sample of 696 families of children with and without a diagnosis of ADHD gave informed consent to participate in the study. Parents of ADHD children were recruited from some child mental health clinics and family support associations. The ADHD sample included parents of 399 children aged 6–18 years (*M* = 11.65; *SD* = 3.1; 280 males). Mental health professionals entirely blinded regarding the objectives of the study, performed the ADHD diagnosis. They all used the inattention (ADHD-IN) and hyperactivity/impulsivity (ADHD-HY) symptoms from the ADHD-Rating Scale-IV (DuPaul et al., 1998) for ADHD diagnosis purpose. From the ADHD group, 307 children were of the combined subtype and 92 from the inattentive subtype. The control group was composed of parents of 297 children aged 6–18 years without ADHD symptoms (*M* = 10.91; *SD* = 3.2; 149 male), recruited from some schools in the local area. Control participants were excluded if they scored above the clinical threshold of ADHD-RS-IV parent ratings. Demographic and clinical characteristics of the whole sample are shown in **Table 1**.

**Table 1:** Demographic and clinical information of clinical and control samples.

				Clinical subgroup		
	Control group	Clinical group		ADHD-combined	ADHD-inattentive	
M(SD)	*n* = 297	*n* = 399	*F*/χ^2^	*n* = 307	*n* = 92	*F*/χ^2^
Age	10.91 (3.2)	11.65 (3.1)	15.66^∗∗^	11.69 (3.0)	12.47 (3.2)	4.65^∗^
Gender (male%)	50.2	70.1	27.18^∗∗^	72.04	63.29	2.25
Medicated (%)		58.4		61.24	48.91	3.28
ADHD-RS-IV						
Inattention	3.95 (3.3)	19.24 (4.8)	2187.3^∗∗^	19.69 (4.7)	17.32 (4.8)	14.28^∗∗^
Hyperactivity/ impulsivity	3.72 (3.2)	15.03 (6.7)	705.97^∗∗^	17.68 (5.0)	6.25 (3.5)	349.42^∗∗^

*>ADHD-RS-IV = ADHD Rating Scale IV. ^∗^*p* < 0.05, ^∗∗^*p* < 0.01.*

The Ethics Committee of the University of Murcia approved the study. All participants were informed of the objectives and methods of the study. Parents completed the clinical scales and a brief sociodemographic questionnaire in web format. We guaranteed confidentiality of participants throughout the study.

### Procedure

The scale construction followed an item improved sequential process. The first pool of items was comprised of 55 items equally distributed along the seven areas. We first selected a committee of four experts from university academics and clinicians specialized in ADHD. The committee assessed the original pool and reduced it from 55 to 20 items by selecting the most comprehensive items on the basis of clarity, precision, and plainness. Those items which were agreed in terms of the precision in their definition and the degree of sufficiency were selected. Thirteen psychology postgraduate and 66 psychopedagogy undergraduate students that volunteered to participate, formed a second group. Students rated each remaining item with a score ranging from 0 to 4 on the basis of their clarity, intelligibility, and ease to understand (e.g., 0 = “Not clear at all” to 4 = “Absolutely clear”). Items reaching average scores of 3 or less, and/or Content Validity Index (i.e., experts’ ratings of item relevance) lower than 0.50 were further excluded, reducing the pool from 20 to 17 items (see **Table 2**).

**Table 2:** Seven areas assessed for the current study by First Scale Model (abbreviated item form).

**Emotional management** (*Emotional Self-regulation*). S/he has difficulties controlling or hiding emotions, especially negative ones (e.g., anger, frustration, sadness, etc.). (*Self-Esteem*). S/he has low self-esteem. (*Emotional lability*). Is s/he emotionally unstable (i.e., easily changes from enthusiasm to discouragement)? (*Restless management*). S/he feels excessive restless some days prior to certain dates (e.g., birthdays, parties, holidays, etc.) (*Sensitive to reward*) Is s/he very sensitive to encouraging words and recognition for his/her achievements? **Fine motor coordination** (*Handwriting*). S/he has very poor handwriting (e.g., omits letters or syllables, or s/he has an irregular spatial arrangement even with guide lines, almost illegible, etc.). (*Handicrafts*). S/he has difficulties in performing handicrafts, which require accuracy and delicacy (e.g., manual arts, crafts, etc.). **Problem solving/management of time** (*Executive Functions*). When s/he deals with a problem, does s/he have difficulties in planning and implementing different steps for solving the problem? (*Management of Time*). S/he has trouble with time management/organization (e.g., fails to submit homework on time, fails judging how much time it will take him/her to do something, etc.). (*Temporal Sequencing*). S/he has difficulty in explaining things or events in their correct order, s/he forgets some details and/or makes chronological inaccuracies. **Disruptive behavior** (*Limits*). S/he has difficulties in understanding where the limits are, and s/he can end up making a game disagreeable and/or unpleasant. **Sleep habits** (*Sleep Habits*). S/he has sleep difficulties (some troubles falling asleep, not rest sufficiently, s/he moves a lot or has breathing problems during sleep). **Academic achievement** (*School Diary*). S/he fails to accurately note down the homework and exams in the school diary (or s/he does it incorrectly or incompletely). (*Academic Support*). S/he requires continued academic support (by a family member, private tutor, etc.). (*Reading Comprehension*). S/he has significant difficulties in reading comprehension. (*Maths*). S/he has significant difficulties in maths. **Quality of life** (*Quality of Life*). Family quality of life has been badly affected by the problems related with your child’s behavior.

*Each item was rated on a 4-point scale (i.e., Not true, A little true, Quite true, and Completely true). The title was: “Please read each item carefully, then mark how well it describes the child’s behavior in the past 6 months.”*

The resulting 17 items of the ADHD-CDS, as well as the rest of questionnaires and scales (sociodemographic, diagnosis, medication, and ADHD-Rating Scale-IV), were fitted into a web format. Parents were given access to the scales online and results were encrypted for later correction. Parents first rated the occurrence of inattention and hyperactivity/impulsivity symptoms affecting their children for the past 6 months on a Likert-type 4-point scale (0 = never or rarely, 1 = sometimes, 2 = often, 3 = very often), according to the ADHD Rating Scale-IV (DuPaul et al., 1998). The two types of ADHD-related symptoms have demonstrated adequate psychometric properties in previous studies with both American and Spanish children (Servera and Cardo, 2007). Cronbach’s alphas for the inattention and hyperactivity/impulsivity subscales for parents’ report were 0.91 and 0.90, respectively. Later on, parents provided sociodemographic and clinical information and filled in the current ADHD-CDS.

### Statistical Analysis

Responses collected from parents to the ADHD-CDS were analyzed according to the following sequence:

(i) Differences between parents’ responses from the clinical and control groups were computed by using non-parametric tests. The Mann–Whitney *U* test was used to compare the control vs. the clinical groups, and the Kruskall–Wallis χ^2^ test was used to compare the control vs. the ADHD clinical presentations. We checked whether case-control differences were due to either age or gender.
(ii) Principal Axis Factoring was used to determine the latent structure of items. Such method of factoring is recommended for non-normal distributions (Costello and Osborne, 2005). We included all participants to maximize statistical power. Alternative models were also compared with the single-factor model, and at this end we forced the rotation.
(iii) Confirmatory Factor Analysis with diagonally weighted least squares method (cat-DWLS) was performed on the basis of the re-specified model from EFA. We used Mplus 14 software for that purpose.
(iv) ADHD-CDS single total scores (i.e., the sum of items) were computed and used to estimate correlations. We also computed unique effects among ADHD-IN, ADHD-HY, and ADHD-CDS total score (Spearman’s correlation and multiple regressions analysis, respectively).
(v) We conducted sensitivity and specificity analyses, and estimated the area under the ROC curve.

## Results

Non-parametric analyses were conducted with the total sample because item-domain scores followed non-normal distributions (Kolmogorov–Smirnov test *p* < 0.05). Concretely, the Mann–Whitney *U* test was employed to assess the differences between the control and the clinical taken gender as a between-subjects factor; and Cliff’s delta was used to estimate the effect sizes. In contrast, the clinical sample scores followed a normal distribution (Kolmogorov–Smirnov test *p* > 0.05).

In the clinical group males outperformed females in handicrafts (*U* = 5124; δ = 0.25), limits (*U* = 5690; δ = 0.17), and school diary (*U* = 5482; δ = 0.20), whereas females outperformed males in mathematics (*U* = 5280; δ = -0.23). The same gender differences were also found in the control group, but the male superiority extended to handwriting (*U* = 3752; δ = 0.15), and quality of life (*U* = 3814; δ = 0.14). Children from the clinical group that were taking medication showed lower scores in both limits (*U* = 5746.5; δ = 0.20) and quality of life (*U* = 5911; δ = 0.22), compared to children that were not taking medication. Age correlated significantly with time management (ρ = 0.17, *p* < 0.01), quality of life (ρ = 0.15, *p* < 0.01), and mathematics (ρ = 0.28, *p* < 0.01).

Both the ADHD-combined and the ADHD-inattentive subgroups showed higher scores than the control group in all domains (see **Table 3**). Nevertheless, some differences between the two clinical subgroups were also found. The ADHD-inattentive subgroup showed lower scores than the ADHD-combined subgroup in emotional self-regulation, emotional lability, handwriting, problem solving, quality of life, and limits.

**Table 3:** Mean scores in the preliminary 17-item scale in both the clinical (and clinical subgroups) and the control groups.

			Clinical subgroup	
	Control group (1)	Clinical group	ADHD-combined	ADHD-inattentive
*M* (*SD*)	*n* = 297	*n* = 399	*n* = 307	*n* = 92
(1) Emotional self-regulation	1.06 (1.04)	2.34 (0.93)	2.45 (0.85)	1.98 (1.08)
(2) Self-esteem	0.39 (0.73)	1.72 (1.03)	1.76 (1.02)	1.77 (1.06)
(3) Emotional lability	0.47 (0.76)	1.79 (1.01)	1.91 (0.97)	1.39 (1.04)
(4) Restless management	1.03 (0.97)	1.90 (0.97)	2.06 (0.92)	1.39 (0.97)
(5) Sensitive to reward	1.53 (0.96)	2.36 (0.80)	2.36 (0.79)	2.36 (0.83)
(6) Handwriting	0.40 (0.80)	1.89 (1.01)	1.98 (1.08)	1.61 (1.17)
(7) Handicrafts	0.48 (0.75)	1.67 (1.01)	1.72 (1.08)	1.51 (1.13)
(8) Problem solving	0.50 (0.63)	2.10 (0.85)	2.16 (0.83)	1.92 (0.87)
(9) Management of time	0.47 (0.71)	2.40 (0.81)	2.39 (0.82)	2.45 (0.76)
(10) Temporal sequencing	0.25 (0.58)	1.74 (1.03)	1.80 (1.03)	1.54 (1.01)
(11) Limits	0.32 (0.62)	1.67 (1.02)	1.85 (0.96)	1.04 (0.97)
(12) Quality of life	0.16 (0.46)	1.85 (1.05)	1.93 (1.01)	1.58 (1.15)
(13) Sleep habits	0.29 (0.65)	1.31 (1.13)	1.44 (1.13)	0.90 (1.05)
(14) School diary	0.34 (0.66)	2.05 (0.98)	2.08 (0.97)	1.95 (1.02)
(15) Academic support	0.41 (0.85)	2.37 (0.91)	2.37 (0.92)	2.38 (0.89)
(16) Reading comprehension	0.33 (0.63)	1.67 (1.01)	1.65 (1.12)	1.74 (1.04)
(17) Maths	0.35 (0.70)	1.45 (1.17)	1.49 (1.12)	1.59 (1.19)

The control group mean scores were greater than 1 in three items, emotional self-regulation, restless management, and sensitive to reward. As the mean scores of those three items were higher than the 0.5 exclusion criterion for controls, and almost three times greater than the mean of the remaining items (*M* = 0.37), they were further excluded from the scale. Additionally, the sleep habits item scored below 1.5 in the clinical group, and thus is was also excluded from the scale.

Spearman’s rank correlation coefficients were also computed (**Table 4**). Correlation between ADHD-dimensions and items were moderate-to-high for inattention (ρ = 0.52 to 0.81; *p*s < 0.001), and low-to-high for hyperactive/impulsivity (ρ = 0.37 to 0.70; *p*s < 0.001). With the exception of limits (ADHD-IN, ρ = 0.68, *p* < 0.001; ADHD-HY ρ = 0.72, *p* < 0.001), the correlations for each domain were greater for ADHD-IN (ρ ranged from 0.52 to 0.81; all *p*s < 0.001) than for ADHD-HY (ρ ranged from 0.34 to 0.72; all *p*s < 0.001).

**Table 4:** Spearman’s correlations between items and ADHD dimensions (*n* = 696).

	Inattention	Hyperactivity/impulsivity
	ρ	ρ
(2) Self-esteem	0.62^∗^	0.48^∗^
(3) Emotional lability	0.62^∗^	0.61^∗^
(6) Handwriting	0.65^∗^	0.58^∗^
(7) Handicrafts	0.57^∗^	0.51^∗^
(8) Problem solving	0.77^∗^	0.64^∗^
(9) Management of time	0.81^∗^	0.63^∗^
(10) Temporal sequencing	0.71^∗^	0.61^∗^
(11) Limits	0.66^∗^	0.70^∗^
(12) Quality of life	0.71^∗^	0.65^∗^
(14) School diary	0.75^∗^	0.60^∗^
(15) Academic support	0.76^∗^	0.59^∗^
(16) Reading comprehension	0.62^∗^	0.47^∗^
(17) Maths^a^	0.52^∗^	0.36^∗^

*^a^Spearman’s partial correlation (maths was correlated with age; ρ = 0.276, *p* < 0.001). ^∗^*p* < 0.001.*

### Factor Structure of ADHD-CDS Items

The results showed a 13-item single factor model with good fit indices (**Table 5**). Factor loadings ranged from 0.62 (maths) to 0.85 (academic support). Results of the 13-item exploratory factor analysis supported a single total score. The sum of 13 items generated an ADHD-CDS total score, with higher scores indicating greater degree of ADHD difficulties (total score ranging from 0 to 39). Cronbach’s alpha coefficient was 0.94, and corrected item-total correlations were medium-to-high, ranging 0.59 to 0.82, indicating high internal consistency reliability.

**Table 5:** Single factor loadings for the 13-items scale (principal axis factoring).

	Loadings
Self-esteem	0.737
Emotional lability	0.706
Handwriting	0.685
Handicrafts	0.608
Problem solving	0.848
Management of time	0.843
Temporal sequencing	0.766
Limits	0.697
Quality of life	0.766
School diary	799
Academic support	0.845
Reading comprehension	0.690
Maths	0.618
KMO	0.952
Eigenvalue	7.183
% Variance	55.25

We also computed two- and three- exploratory factor models by forcing the rotations. However, because the eigenvalues were significantly lower than 1 (eigenvalues from 0.487 to 0.502) the multi-factor models were rejected (Kaiser’s criterion). Accordingly, we did not conduct any confirmatory factor analysis with the alternative models, nor did compare them with the single factor model.

An additional confirmatory factor analysis with diagonally weighted least squares method (cat-DWLS) was also conducted to verify that each item loaded onto one single component factor. All items converged into one general factor, ADHD concomitant difficulties, with χ^2^ (65) = 543.36; *p* < 0.001; RMSA = 0.01; WRMR = 1.586; CFI = 0.98; TLI = 0.98. Standardized factor loadings were from 0.61 to 0.91. Hence, the fit indices indicated a satisfactory fit to the single-factor structure^1^.

### Correlations and Unique Effects among ADHD-IN, ADHD-HY and ADHD-CDS Total Score

We computed Spearman rank correlation coefficients between the ADHD dimensions and the total score. Total score was positive correlated with both ADHD-IN (ρ = 0.88; *p* < 0.001) and ADHD-HY (ρ = 0.74; *p* < 0.001). Nevertheless, when only the clinical sample was analyzed, the correlations were rather moderate (for ADHD-IN, ρ = 0.541; *p* < 0.001; for ADHD-HY, ρ = 0.345; *p* < 0.001). After controlling for ADHD-IN there was no relationship between ADHD-HY and ADHD-CDS (β = 0.05; *SE* = 0.06; *p* > 0.05). The ADHD-IN scores predicted ADHD-CDS scores for both the clinical sample (β = 0.50; *SE* = 0.08; *p* < 0.001) and the whole sample (β = 0.85; *SE* = 0.01; *p* < 0.001).

### ROC Curve Analysis

The ROC curve for the ADHD-CDS (against the ADHD diagnostic status) gave an area under the curve (AUC) of 0.979 (95%, CI = [0.969, 0.989]), which can be considered very high (Sweet, 1988). The AUC was 0.980 (95%, CI = [0.967, 0.994]) for males, and 0.969 (95%, CI = [0.945, 0.992]) for females. ROC curve analyses were also conducted differentiating between the two clinical subgroups. According to the clinical diagnosis, AUC was 0.981 (95%, CI = [0.970, 0.992]) for the ADHD-combined subgroup and 0.974 (95%, CI = [0.959, 0.989]) for the ADHD-inattentive subgroup.

In addition, the percentage of ADHD cases that scored higher than the 90% of the control group scores was 94.3%. Differentiating between the two clinical subgroups, the percentages were 95.4 and 89.2% for the ADHD-combined and the ADHD-inattentive subgroups, respectively.

## Discussion

It is clear that ADHD is a clinical and neuropsychological heterogeneous disorder. At the clinical level, the two main ADHD dimensions described in DSM (i.e., inattention and hyperactivity/impulsivity) are widely validated, although the bidimensional model of ADHD has been recently questioned (Parke et al., 2016). Further, the validity of the three nominal subtypes (i.e., the predominantly hyperactive/impulsive subtype, the predominantly inattentive subtype, and the combined subtype) is also under debate (Willcutt et al., 2012). The debate extends to the relationship between Sluggish Cognitive Tempo (SCT) and ADHD, questioning whether SCT fits well or not into the ADHD DSM model (Willcutt et al., 2014). These are good examples that suggest that the ADHD diagnosis is constantly being reviewed and updated.

At the neuropsychological level, multiple neurocognitive deficits have been associated with the disease. Delay aversion, inhibitory control, timing, time variability, decision-making, and working memory among others, are crucial neuropsychological areas that have been found to be altered in ADHD (e.g., Sonuga-Barke, 2003; Sonuga-Barke et al., 2010; de Zeeuw et al., 2012). Apart from the core clinical and neuropsychological deficits, individuals with ADHD usually show other concomitant difficulties, which are not solely related with ADHD. The nature of the relationship between ADHD and these concomitant difficulties is still unclear, but their high frequency of co-occurrence should be taken into consideration. Thus, an appropriate assessment of those concomitant difficulties associated with ADHD is of special relevance for future research and clinical practice.

In the present study we aimed at constructing a brief scale, the ADHD-CDS, that may be a useful and easy-to-use instrument to detect comorbidity associated to ADHD in both clinical and research contexts. These difficulties might also be the target of clinical interventions concerned with ADHD, such as behavioral modification therapy, emotional and motivational self-management skills, family therapy, and/or metacognitive strategies among others.

Regarding the ADHD-CDS structure, our results with a rather ample sample of both clinical and control participants, suggest that our scale follows a single-factor latent structure. Single-factor models have also been observed in other screening scales when they have been used in both clinical and non-clinical populations (Gomez et al., 2003, 2005). In addition, the present ADHD-CDS shows a high potential discriminatory value for screening ADHD profiles. The predictive value of ADHD-CDS is related to inattention symptoms (but not to hyperactivity/impulsivity symptoms), which allows us to discriminate ADHD profiles irrespective of their clinical subtypes. Thus, ADHD-CDS represents an important improvement from previous ADHD screening scales (e.g., the Strengths and Difficulties Questionnaire; Goodman, 1997), which seem to be influenced by clinical subtypes (e.g., Ullebø et al., 2011).

Finally, the present study has several limitations. First, ADHD-CDS assesses ADHD concomitant difficulties, and thus we cannot establish any causal relation between such deficits and the disorder. Second, as other disorders have not been included in the study we cannot assure that the impairment profile obtained with ADHD-CDS is unique to ADHD. Third, ADHD-CDS includes some but not all concomitant difficulties that may be associated with ADHD. Thus, the current scale should be considered as a preliminary proposal, which is open to the inclusion of other ADHD concomitant difficulties that clinicians may consider relevant in the diagnosis and treatment of ADHD. Four, from a methodological perspective, further studies are needed to test the psychometric properties of the scale on independent samples, particularly including people with other disorders different to ADHD (i.e., ODD, Autism Spectrum Disorders, Intellectual Disability).

In summary, the present results provide additional evidence that ADHD is a complex and highly heterogeneous disorder with some concomitant difficulties in several functional areas. The ADHD-CDS has shown preliminary adequate psychometric properties, with high convergent validity and good sensitivity for different ADHD profiles, which makes it a potentially appropriate and brief instrument that may be easily used by clinicians, researchers, and health professionals in dealing with ADHD.

## Author Contributions

JF-C was involved all along the process, from the conception to the revision to the manuscript. LF was involved in the drafting and revision of the manuscript.

## Conflict of Interest Statement

The authors declare that the research was conducted in the absence of any commercial or financial relationships that could be construed as a potential conflict of interest.

## Footnotes

1 The ADHD sample includes 70% of males, a figure that fits well with the proportion of males compared with females that are diagnosed with ADHD. However, the control group sample size reflects what it should be expected by random selection. To rule out the possibility that the differences between the ADHD and control groups are due to differences in gender proportion (and probably sample size) in the two samples, we re-ran the analyses with a reduced sample, randomly chosen, from both the clinical and control groups, so that both gender proportion (fitting the male proportion of ADHD diagnosis) and sample size were matched between the two groups (*n* = 256; 71.8% males). The results also showed a 13-item single factor model with good fit indices, both for EFA (KMO = 0.953; *p* < 0.001; eigenvalue = 7.30; 56% variance explained; loads 0.62 to 0.86 and CFA [χ^2^ (65) = 378.32; *p* < 0.001; RMSA = 0.09; WRMR = 1.325; CFI = 0.98; TLI = 0.98. Standardized factor loadings were from 0.68 to 0.92]. The reliability was high (Cronbach’s alpha = 0.94). There were significant differences between the clinical and the control group for ADHD-CDS total score (Mann–Whitney *U* = 1695; *p* < 0.001 or *T*-test = -34.62; *p* < 0.001). The correlations between ADHD dimensions and ADHD-CDS total score were high (Spearman’s Rho = 0.88 for inattention and 0.76 for hyperactivity/impulsivity).
